# Stress, glucocorticoid hormones, and hippocampal neural progenitor cells: implications to mood disorders

**DOI:** 10.3389/fphys.2015.00230

**Published:** 2015-08-19

**Authors:** Tomoshige Kino

**Affiliations:** Division of Experimental Biology, Department of Experimental Therapeutics, Sidra Medical and Research CenterDoha, Qatar

**Keywords:** dentate gyrus, glucocorticoid receptor, hippocampus, major depression, neural stem cells (NSCs)

## Abstract

The hypothalamic-pituitary-adrenal (HPA) axis and its end-effectors glucocorticoid hormones play central roles in the adaptive response to numerous stressors that can be either internal or external. Thus, this system has a strong impact on the brain hippocampus and its major functions, such as cognition, memory as well as behavior, and mood. The hippocampal area of the adult brain contains neural stem cells or more committed neural progenitor cells, which retain throughout the human life the ability of self-renewal and to differentiate into multiple neural cell lineages, such as neurons, astrocytes, and oligodendrocytes. Importantly, these characteristic cells contribute significantly to the above-indicated functions of the hippocampus, while various stressors and glucocorticoids influence proliferation, differentiation, and fate of these cells. This review offers an overview of the current understanding on the interactions between the HPA axis/glucocorticoid stress-responsive system and hippocampal neural progenitor cells by focusing on the actions of glucocorticoids. Also addressed is a further discussion on the implications of such interactions to the pathophysiology of mood disorders.

## Introduction

Humans face in their daily activities unpredicted short- and long-term stressful events called stressors, which are either internal (e.g., hurtful memories, internal injuries, neoplasias) or external (e.g., pathogen infection, excessive heat or cold, food deprivation, trauma, and invasive pathogens) (Chrousos, 2009). To cope with such threats, the central nervous system (CNS) integrates the information sensed by peripheral organs, and adjusts the body's activities through its effector machinery called the stress system. Among this system, the hypothalamic-pituitary-adrenal (HPA) axis and its end-effectors glucocorticoid hormones play central roles by cooperating with another important axis, the locus ceruleous/norepinephrine-autonomic systems and their end-products, norepinephrine and epinephrine (Chrousos, 2009). This stress system restores internal homeostasis by regulating many biological activities, including those of the CNS (Chrousos and Kino, 2007; Chrousos, 2009). However, this system is sometimes unrestrained and maladaptive if the stress stimulus is of a quality, intensity and/or chronicity that exceeds the threshold of the individual's ability to effectively manage, resulting in the development of an array of adverse effects, such as mood alteration, induction of anxiety, and cognitive dysfunction, as well as immune suppression, osteoporosis, and central obesity-associated insulin resistance and hyperlipidaemia (Kino et al., 2003; Chrousos and Kino, 2007; Chrousos, 2009).

The brain hippocampus and its associated dentate gyrus, part of the limbic system located in the temporal lobe, consist of the CNS circuitry that organizes adaptive response to stress through communicating with other brain areas, such as the paraventricular nucleus (PVN) of the hypothalamus and prefrontal cortex (Jonas and Lisman, 2014). In addition to participating in the stress response, the hippocampus and the dentate gyrus play an important role in the regulation of cognition, learning, short- to long-term memory consolidation, special navigation, and mood (Gold et al., 1988a,b; Jonas and Lisman, 2014). Recently, neural stem cells (NSCs) or neural progenitor cells that retain a potential of self-renewal and an ability to differentiate into multiple neural component cells, are identified in the hippocampal dentate gyrus. These two types of specialized neural cells are similar but different in that progenitor cells are more committed “stem cells” with a tendency to differentiate into specific types of cells and a replication potential for a limited number of times (Androutsellis-Theotokis et al., 2008a). Hippocampal NSCs or progenitor cells participate in learning/memory and to the individual's response to anti-depressants and the subsequent recovery from the depressive state (Shors, 2008; Zhao et al., 2008; Anacker et al., 2011). Importantly, glucocorticoids influence their activities, which may underlie the adverse effects of these steroids on hippocampal functions, such as memory, cognition, and mood (Kino and Chrousos, 2004a). This review will explain the current understanding of hippocampal NSCs or progenitor cells, and discuss the influence that stress and glucocorticoid hormones have on these particular cell groups, and the potential roles of their interaction in the pathophysiology of mood disorders.

## Neural progenitor cells in adult hippocampal dentate gyrus

NSCs are defined by their properties of self-renewal and the ability to differentiate into multiple neural cell lineages, such as neurons, astrocytes, and oligodendrocytes. NSCs also play central roles in the development of the fetal CNS acting as sources of neural component cells (Androutsellis-Theotokis et al., 2008a,b). Differentiated neurons are functional components for organizing the complex neuronal circuits critical for brain functions, such as processing of information and transmission, while astrocytes and oligodendrocytes are known as glial cells and support proper functioning of the neurons (Androutsellis-Theotokis et al., 2008a). In the adult brain, NSCs are present in some specific brain areas among which the dentate gyrus of the hippocampal formation is the most well-known place for harboring these cells (Androutsellis-Theotokis et al., 2008a). NSCs are also found in the rodent subventricular zone lining the lateral ventricules, and these cells provide neurons to the olfactory bulb for supporting the olfaction activity through their migration along the rostral migratory stream (Zhao et al., 2008). In addition to these two well-established brain areas harboring NSCs, other brain portions containing NSCs have been increasingly reported (Androutsellis-Theotokis et al., 2009). The putative progenitor cells are even found in the adult adrenal medulla, and these cells share some characteristics of NSCs, suggesting that NSCs or their related cells may also be present in this peripheral neuronal tissue (Androutsellis-Theotokis et al., 2012; Bornstein et al., 2012).

**Figure 1:**
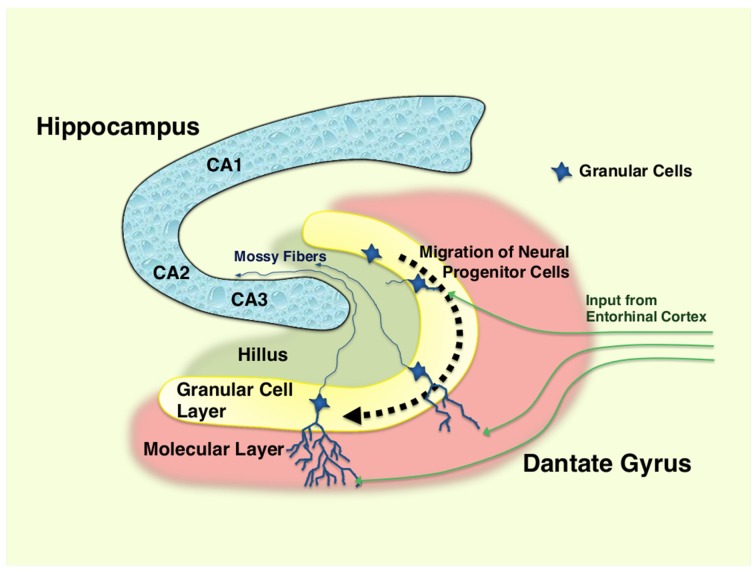
**Hippocampus and differentiation of neural progenitor cells in dentate gyrus**. Neural progenitor cells reside in the granular layer (subgranular zone) of the dentate gyrus as a mixture with immature and mature granular cell neurons. Following the differentiation (~1–2 months), immature neurons generated from neural progenitor cells migrate into the lower arm of the dentate gyrus, and extend their dendrites into the molecular layer, which receives excitatory neurons from the entorhinal cortex. They also spread into the hillus their excitatory mossy fibers, and form synaptic connections to the pyramidal neurons in the CA3 region of the hippocampus. Over half of the immature neurons die during their differentiation process. CA1, 2, and 3: region 1, 2, and 3 of the hippocampal proper.

In the adult dentate gyrus, NSCs are consistently found throughout the human life as highly polarized excitatory cells of the granular cell layer (subgranular zone) in a mixture with the immature newborn and mature neurons (Zhao et al., 2008) (Figure 1). These adult NSCs or hippocampal progenitor cells are identified as granule cells in this portion of the brain, and they retain an ability to continuously proliferate to maintain their population in the neurogenic niche, which is mainly formed by residential astrocytes and glutamate supply from the vasculature in the dentate gyrus (Zhao et al., 2008). In addition, thousands of new cells committed to the neuronal lineage are produced from hippocampal progenitor cells every day, but over half (~60%) of these cells die during the first 1–2 weeks (Gould et al., 1999). Newly generated and surviving immature neurons migrate short distances into the lower arm of the granule cell layer and extend the output unmyelinated axons, called mossy fibers, to the pyramidal cells of the hippocampal CA3 area during the first week after birth (Shors, 2008). During the next several weeks, these cells mature into functional neurons producing action potentials and are incorporated into the functional network through formation of synaptic connections to inhibitory basket cells, interneurons and excitatory mossy cells residing in the hippocampus, in addition to the pyramidal cells located in its CA3 region (Zhao et al., 2008; Jonas and Lisman, 2014). Upon maturation, these cells also extend dendrites covered with spines into the molecular layer of the dentate gyrus and receive input signals from the entorhinal cortex, which represents the majority of excitatory synapses on these cells (Zhao et al., 2008).

## Neural progenitor cells and hippocampal functions

The hippocampus and the dentate gyrus play substantial roles in the learning and the memory formation (Smith and Bulkin, 2014; Spiers and Bendor, 2014). It is therefore speculated that adult neural progenitor cells and their derived granular cell neurons participate in these brain activities. Indeed, the learning task associated with hippocampus-mediated memory formation increases the number of granular cells in the dentate gyrus, indicating that learning is a positive factor for neuronal progenitor cells possibly rescuing their differentiated form from cell death (Shors, 2008). In contrast to the above finding indicating involvement of hippocampal progenitor cells in some learning tasks, other reports have suggested no roles of these cells in learning tasks in animals where anti-mitogenic agents were used to avoid cell proliferation (Shors, 2008). However, conclusive evidence in one recent report showed that increasing neurogenesis in the hippocampal dentate gyrus is sufficient for improving pattern separation, an activity of disambiguate a new experience from stored memories (Sahay et al., 2011). Thus, adult neural progenitor cells play roles in some hippocampus-mediated memory consolidation/preservation and its subsequent organization.

Another important role of the hippocampus is the regulation of behavior and mood (Jonas and Lisman, 2014). Involvement of adult hippocampal progenitor cells in behavior and mood is more complicated than their roles in learning and memory, but it was first suggested by the finding obtained with anti-depressants: these compounds acting through the monoamine system increase newly generated neurons in the dentate gyrus (Surget et al., 2011). Indeed, it usually takes 3–4 weeks for anti-depressants to exert their pharmacologic (anti-depressive) effect, even though they change monoamine levels within several hours (Surget et al., 2011). Since neurogenesis also takes 3–4 weeks to develop mature neurons in the dentate gyrus (Zhao et al., 2008), the similar time-course suggests a potential of hippocampal progenitor cells in supporting the actions of anti-depressants, and further, in the regulation of behavior and mood by the hippocampus. This promising hypothesis was subsequently tested in several experimental systems with controversial results, which indicate neural remodeling or recruitment of new neurons play more important roles in mediating the actions of anti-depressants than their effects of stimulating neurogenesis (Bessa et al., 2009; Surget et al., 2011). However, the hippocampal neurogenesis was finally shown to be sufficient for reducing anxiety and depression-like behavior by using the mice expressing the pro-apoptotic Bax, which specifically deletes neural progenitor cells in the dentate gyrus (Hill et al., 2015). The subsequent finding that hippocampal neurogenesis was required for pattern separation further suggested that adult neural progenitor cells residing in hippocampus participate in the mood change by stress, as this brain activity is frequently damaged in patients with post-traumatic stress disorder (PTSD) (Sahay et al., 2011).

Hippocampal neurogenesis was also shown to be critical for the hippocampus-mediated negative control on the HPA axis by using the animals whose hippocampal neurogenesis was inhibited either by radiation or transgenic approach (Snyder et al., 2011).

## Hypothalamic-pituitary-adrenal (HPA) axis, glucocorticoids, and glucocorticoid receptor

**Figure 2:**
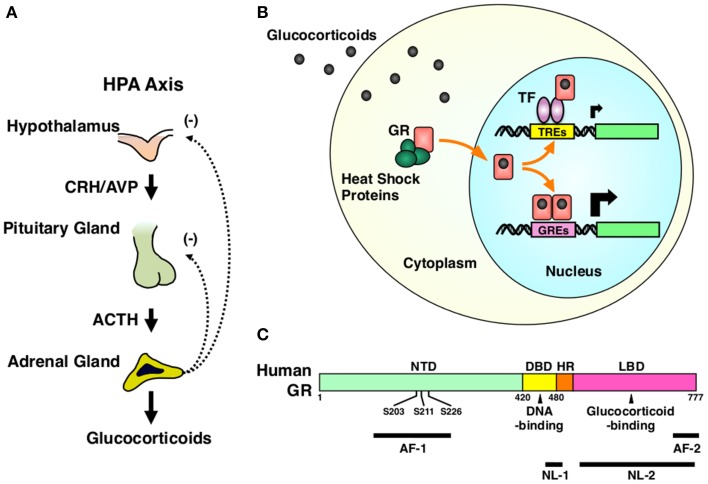
**HPA axis and GR. (A)** Organization of the HPA axis. The HPA axis consists of three components, PVN of the hypothalamus, the anterior pituitary gland, and the adrenal cortex. Neurons residing in PVN produce CRH and AVP, and release them into the pituitary portal system under the control of the upper regulatory centers, such as the central circadian rhythm center hypothalamic suprachiasmatic nucleus (SCN) and the stress responsive hippocampus, prefrontal cortex, and the amygdala. CRH and AVP stimulate the secretion of ACTH from the corticotrophs of the anterior pituitary gland. Circulating ACTH then stimulates the production and the secretion of glucocorticoids (cortisol in humans and corticosterone in rodents) from the adrenocortical cells located in the *zona fasciculata* of the adrenal gland. Secreted glucocorticoids then suppress the upper regulatory centers including PVN and the pituitary gland, forming a closed regulatory loop. **(B)** Intracellular circulation of the GR. In the absence of glucocorticoids, GR resides in the cytoplasm forming a heterocomplex with several heat shock proteins (HSP), including HSP90, 70, and 23. Upon binding to glucocorticoids, GR releases HSPs, exposes its nuclear localization signals (NLs) to the nuclear pore complex and translocates into the nucleus. In the nucleus, GR directly binds as a homodimer its specific recognition sequences called glucocorticoid response elements (GREs), which are located in the promoter region of glucocorticoid-responsive genes, and stimulates their transcriptional activity by attracting many transcriptional cofactors and the RNA polymerase II complex. GR also modulates the transcriptional activity of other transcription factors through physical protein-protein interactions without associating directly to DNA. After regulating the transcription of glucocorticoid-responsive genes, GR moves back into the cytoplasm with the help of the nuclear export system and returns to its ligand friendly condition by reforming a heterocomplex with HSPs. **(C)** Linearized protein structure of the human GR and its functional distribution. Human GR consists of 777 amino acids and composes of three subdomains, the N-terminal or immunogenic domain (NTD), middle DNA-binding domain (DBD), and the C-terminal ligand-binding domain (LBD). Between DBD and LBD, there is a small area called the hinge region (HR). GR has two transactivation domains, activation function (AF)-1 and -2, which are respectively located in NTD and LBD. GR also has two nuclear localization signals (NL)-1 and -2. NL-1 is located in the DBD-HR boarder and mediates the rapid nuclear translocation of GR by communicating with the importin α/β nuclear pore complex, while NL-2 distributes in the entire LBD and mediates slow nuclear translocation of this receptor. GR has three serine residues (serines 203, 211, and 226) in the AF-1 of NTD, which are phosphorylated by several serine/threonine-directed protein kinases including CDK5. ACTH, adrenocorticotropic hormone; AF-1 and -2, activation function-1 and -2; AVP, arginine vasopressin; CRH, corticotropin-releasing hormone; DBD, DNA-binding domain; GR, glucocorticoid receptor; GREs, glucocorticoid response elements; HPA axis, hypothalamic-pituitary-adrenal axis; HR, hinge region; HSPs, heat shock proteins; LBD, ligand-binding domain; NL-1 and -2, nuclear localization signal-1 and -2; NTD, N-terminal domain; S203, 211, and 226, serine at amino acid position 203, 211, and 226; TF, transcription factor; TREs, transcription factor response elements.

The HPA axis consists of three components, the hypothalamic paraventricular nucleus (PVN), the pituitary gland and the cortex of adrenal glands (Kino and Chrousos, 2004b; Nader et al., 2010). Hypothalamic PVN is a central component and contains the neurons that secrete the corticotropin-releasing hormone (CRH) or arginine vasopressin (AVP) (Chrousos, 2009). These peptides are liberated from the median eminence of the hypothalamus into the pituitary portal system and stimulate the secretion of the adrenocorticotropic hormone (ACTH) from the corticotrophs that reside in the anterior pituitary gland. Circulating ACTH then reaches to the *zona fasciculata* of the adrenal cortex and stimulates both production and secretion of glucocorticoids (Chrousos, 2009) (Figure 2A).

Hypothalamic PVN receives many axons from various areas of the brains, such as hippocampus, amygdala, prefrontal cortex, and locus ceruleus of the brainstem, and integrates stressful information obtained from peripheral sensory organs and nerves and then assembled in these brain regions (Gold and Chrousos, 2002). In a basal state, the HPA axis demonstrates circadian activity under the control of the circadian rhythm center suprachiasmatic nucleus (SCN) of the hypothalamus, and creates a typical diurnal fluctuation in serum cortisol concentrations, which reaches the zenith in early morning and the nadir at midnight (Nader et al., 2010; Kino, 2012; Nicolaides et al., 2014). When exposed to stressors, the HPA axis escapes from this regular circadian control and secretes massive amounts of glucocorticoids (cortisol in humans and corticosterone in rodents) to adjust the body's functions (Kino, 2012). After responding to stressors, the HPA axis resets its activity and returns to its normal tone (Chrousos, 2009). However, if the stress exceeds its permissive levels or persists chronically, the HPA axis is continuously activated and high amounts of glucocorticoids are constantly secreted, resulting in the development of an array of problems both in the CNS and peripheral organs (Chrousos and Kino, 2007).

The glucocorticoid receptor (GR), a member of the steroid/thyroid/nuclear receptor superfamily, acts as a tissue receptor for glucocorticoids virtually in all organs and tissues including the CNS, and mediates the actions of these hormones in local tissues (Kino, 2000). GR is a ligand-dependent transcription factor. Upon binding glucocorticoids, GR translocates into the nucleus, binds specific DNA sequences called glucocorticoid response elements (GREs) located in the promoter region of glucocorticoid-responsive genes and changes their transcriptional rates positively or negatively by communicating with numerous transcriptional regulatory molecules, including transcriptional cofactors and the RNA polymerase II (Kino, 2000; Kino et al., 2003). GR also modulates the transcriptional activity of other transcription factors, such as the nuclear factor of κB (NFκB), activator prorein-1 (AP-1) and CRE-binding protein (CREB), through physical protein-protein interactions to these transcription factors without directly associating with DNA (Kino, 2000; Figure 2B). GR consists of three subdomains, N-terminal (NTD), middle DNA-binding (DBD), and C-terminal ligand-binding domain (LBD) (Chrousos and Kino, 2005; Figure 2C). Transcriptional activity of GR is regulated through a number of post-translational modifications, including phosphorylation, acetylation, SUMOylation, and ubiquitylation, to adjust its diverse actions to needs of the local organs and tissues (Kino et al., 2003; Chrousos and Kino, 2005; Kino and Chrousos, 2011; Kino, 2007). Indeed, we previously identified that the “brain specific” cyclin dependent kinase 5 (CDK5), a kinase essential for fetal brain development, maturation of neurons, axon extension, and synaptic activity as well as development of neurodegenerative disorders, phosphorylates the GR and the mineralocorticoid receptor (MR) at serines or threonines located in their N-terminal domain and modulates their transcriptional activity positively or negatively in a gene-specific fashion (Kino et al., 2007, 2010) (Figure 2C).

MR, the closest family member to GR, also functions as a “glucocorticoid receptor” in the hippocampus (Joels, 2008; Le Menuet and Lombés, 2014). MR can bind glucocorticoids with high affinity and serum concentrations of cortisol are over 10-times higher than that of mineralocorticoid aldosterone in humans, thus its specific actions as a signaling mediator for mineralocorticoids in their major target organs, such as kidney collecting tubules and sweat glands, are enabled by the co-expression of the 11β-hydroxysteroid dehydrogenase type 2 (11βHSD2), an enzyme catalyzing active cortisol into inactive cortisone (Wyrwoll et al., 2011). In contrast to GR that is expressed throughout the CNS, MR is found in limited brain areas including the hippocampus (Wyrwoll et al., 2011). This brain area does not express 11βHSD2, thus MR can act as a high affinity receptor for glucocorticoids there (Wyrwoll et al., 2011). Since MR has much higher affinity to glucocorticoids than GR, it is speculated that MR is responsible for mediating the beneficial actions of physiologic levels of glucocorticoids, while GR can mediate those of highly elevated (and sometimes toxic) levels of circulating glucocorticoids observed upon prolonged stress exposure or treatment with these compounds (Sousa and Almeida, 2002). Indeed, high levels of glucocorticoids mediated by GR can cause apoptosis in hippocampal neurons, while activation of MR with its specific agonist aldosterone reverses this adverse action of glucocorticoids (Crochemore et al., 2005).

## Effects of stress and glucocorticoids on hippocampal neural progenitor cells

### Three major functional targets for controlling neurogenesis of the hippocampal progenitor cells

Hippocampal progenitor cells maintain their lineage by self-renewal/proliferation, while they continuously differentiate and mature into neurons and other neural cell components. In addition, neural progenitor cells develop apoptosis and a majority of these cells die during their differentiation into neurons in the hippocampus. Thus, most of the regulatory activities toward hippocampal neurogenesis target these three major activities of the neural progenitor cells: proliferation, differentiation, and cell death/survival (Shors, 2008; Zhao et al., 2008).

### Acute and chronic stress

There are many reports linking stress and reduced hippocampal neurogenesis, and most of these reports support the idea that acute exposure to stress decreases proliferation of neural progenitor cells in the dentate gyrus (Schoenfeld and Gould, 2013). This is evident with multiple stressful stimuli and in several animals including rats, mice, and marmosets. A decrease of neuronal differentiation has also been reported in acute social defeat and acute predator odor exposure in rats (Tanapat et al., 2001; Thomas et al., 2007). Chronic stress reduces hippocampal neurogenesis in both rats and mice as well (Schoenfeld and Gould, 2013). Chronic electronic shock and chronic restraint stress not only reduce progenitor cell proliferation but also suppress neuronal differentiation and cell survival (Schoenfeld and Gould, 2013). The learning paradigm has biphasic effects on hippocampal neurogenesis: it is generally stimulatory, but it becomes suppressive once the learning is complex or prolonged (Gould et al., 1999; Leuner et al., 2004; Aztiria et al., 2007; Dupret et al., 2007), suggesting that unacceptable stress is harmful to hippocampal progenitor cells, decreasing proliferation, differentiation, and/or cell survival. In addition to these direct effects of acute and chronic stress on hippocampal progenitor cells of affected animals, one study showed that prenatal stress, which comes via the mother and impacts the fetus *in utero*, damages the brain development of littermates during its fetal phase and changes behavior in later adult life by causing lifespan reduction of neurogenesis in the dentate gyrus and by blocking learning-induced neurogenesis in this brain area (Lemaire et al., 2000). These results indicate that stress not only affects adult hippocampal neurogenesis in the affected animals, but also has a trans-generation effect, causing life-long effects in littermates when the pregnant mother is exposed to stress.

### Glucocorticoids

As glucocorticoids act as end-effectors for the HPA axis, injection of these hormones in animals overall exerts the effects overlapping with those induced by stress exposure on hippocampal progenitor cells, yet they develop distinct actions, particularly when neural progenitor cells are separated from surrounding tissues and tested *in vitro*. Exogenous administration of corticosterone decreases proliferation and survival of hippocampal progenitor cells in several animal species (Cameron and Gould, 1994; Wong and Herbert, 2006; Lau et al., 2007; Brummelte and Galea, 2010; Yu et al., 2010; Schoenfeld and Gould, 2013). Injection of glucocorticoids in rats also induces apoptosis both in neural progenitor cells and in immature granular cell neurons of their dentate gyri (Yu et al., 2010). Thus, excess amounts of glucocorticoids act as negative regulators for the function/activity of hippocampal progenitor cells *in vivo*, and may mediate in some part the effects of stressful stimuli on these cells.

It is known that the MR agonist aldosterone enhances proliferation of hippocampal progenitor cells in the rats under adrenalectomy, a procedure for eliminating endogenous adrenal steroids including glucocorticoids and mineralocorticoids (Fischer et al., 2002). This suggests that physiologic amounts of glucocorticoids whose effects are mainly mediated by MR in the hippocampus appears to be beneficial and supportive for hippocampal progenitor cells, in contrast to the excess glucocorticoids observed upon stress stimuli or with pharmacologic treatment that are detrimental to them. This finding is further supported by the evidence that the adrenalectomizd rats develop massive loss of granular cells in the dentate gyrus, and this change can be reversed by the administration of aldosterone, a mineralocorticoid that binds with the MR, but not the GR (Woolley et al., 1991). The proposed beneficial effect of physiologic levels of glucocorticoids on hippocampal progenitor cells mediated by MR appears to be an indirect effect through acting on surrounding tissues or compounds/neurotransmitters secreted from distant neurons/cells, as hippocampal progenitor cells and neural stem cells express virtually no MR, although MR levels rapidly and significantly elevate during their differentiation process (Crochemore et al., 2005; Androutsellis-Theotokis et al., 2013). It is also possible that the actions of high-level glucocorticoids may be in some part indirect as well. Indeed, destruction of the entorhinal cortex, one of the major brain areas that extend afferent axons to the dentate gyrus, stimulates production of new neurons in the hippocampus, while blocking the NMDA (N-methyl-D-aspartate) or glutamate receptor that functions in memory and learning and mediates signals for perforant path-dentate gyrus granule cell synapses increases neurogenesis in the hippocampus (Zhao et al., 2008). Further, the brain-derived neurotrophic factor stimulates neurogenesis in the hippocampus, while high doses of glucocorticoids inhibit production of this factor (Kino et al., 2010; Gray et al., 2013). Thus, glucocorticoids could in part influence hippocampal neural progenitor cells indirectly through modulation of these systems and the molecule in addition to their direct effects through the resident GR and MR.

The above-explained results obtained in live animals are important for elucidating the overall influence of glucocorticoids on hippocampal neurogenesis, and provide the strong link to human physiology and pathophysiology. However, these results also harbor internal complexity and sometimes provide inconsistent data between studies most probably due to differences in animal conditions, such as strains, age, sex, and food. Further, multiple biological pathways operate in animals, therefore qualitative and/or quantitative alteration of their inputs to hippocampal progenitor cells may sometimes cause different responses. Thus, *ex vivo* or simple cellular systems are indeed necessary to evaluate direct effects of glucocorticoids on these cells to complement the results obtained *in vivo*.

In line with the requirement of *in vitro* studies evaluating direct effects of glucocorticoids on neural progenitor cells or NSCs, hippocampal progenitor cells were isolated and the effect of glucocorticoids was tested on these cells *in vitro*. The results demonstrate that glucocorticoids are overall suppressive to these cells, and the involvement of the glycogen synthase kinase-3β, β-catenin/transcription factor (TCF) pathway and ubiquitin-mediated degradation of cyclin D1 was suggested in glucocorticoid-induced suppression of their proliferation as potential underlying mechanisms (Sundberg et al., 2006; Boku et al., 2009). We also performed a similar examination in the mouse cortical NSCs by treating them with the synthetic glucocorticoid dexamethasone and by examining its effect on their proliferation and course of differentiation initiated by withdrawal of the fibroblast growth factor-2 (FGF2) (Androutsellis-Theotokis et al., 2008a, 2013). We found that dexamethasone increased the proliferation of NSCs, but also facilitated their differentiation into neurons and astrocytes upon FGF2 withdrawal. Treatment with RU 486, a competitive GR antagonist, efficiently suppressed dexamethasone-induced facilitation of differentiation, indicating that this effect is mediated by the GR expressed in NSCs. Modulation of the Hedgehog signaling pathway by glucocorticoids may be one potential mechanism underlying the glucocorticoid-induced NSC proliferation we observed. Some glucocorticoids (halcinonide, fluticasone, clobetasol, and flucinonide) activate the Hedgehog pathway and induce cell proliferation by stimulating the smoothened (Smo), a membrane receptor for this signaling system (Wang et al., 2010). Discrepancy of our results to the reported *in vivo* effects of glucocorticoids indicates that the effect of glucocorticoids on hippocampal progenitor cells *in vivo* may be a sum of multiple effects, such as those with beneficial effects acting on these cells, and others with adverse effects, for example, influencing directly on these cells or through the surrounding neurogenic niche (Zhao et al., 2008), as we discussed in the *in vivo* effects of glucocorticoids on these cells.

In our experiment, dexamethasone caused massive induction of neurite extension in differentiating cells (Androutsellis-Theotokis et al., 2013). CDK5, the kinase we previously discovered to phosphorylate GR and regulate its transcriptional activity, is required for hippocampal neurogenesis, particularly for the proper extension of dendrites during differentiation of hippocampal progenitor cells (Jessberger et al., 2008). Since acute and chronic stress differentially modulate CDK5 kinase activity in the hippocampus (Papadopoulou et al., 2015), it is possible that CDK5 and ligand-activated GR coordinately regulate dendrite development of the immature granular cells in the dentate gyrus, further contributing to the altered neurogenesis observed upon stress exposure.

## Implications of stress/glucocorticoid-mediated regulation of hippocampal neurogenesis to mood disorders

Chronic stress is a precipitating factor for major depression and significantly influences its disease course. Exposure to chronic stress frequently increases serum cortisol concentrations in human subjects, particularly during the evening (Gold and Chrousos, 2002; Chrousos, 2009). In experimental conditions, stress, and glucocorticoids have strong effects on hippocampal progenitor cells by modulating their proliferation, differentiation and cell fate (Schoenfeld and Gould, 2013). In the clinic, patients with Cushing syndrome who harbor elevated serum cortisol levels due to cortisol- or ACTH-producing neoplasmas develop neurological deficits, such as cognitive and mood disturbances, and shrinkage of their hippocampi (Starkman et al., 1992). Further, patients with major depression develop hippocampal atrophy, while the model animals for depression demonstrate reduced numbers of hippocampal progenitor cells (Sheline et al., 1996). All the above findings suggest that stress and glucocorticoids may contribute to the pathophysiology of major depression by altering hippocampal neurogenesis.

Important research verifying this hypothesis was again shown from the examination on the effect of the anti-depressants to hippocampal neurogenesis (Malberg et al., 2000). In an *in vitro* condition, the popular anti-depressant fluoxetine attenuated the inhibitory effect of glucocorticoids on neurogenesis via a potassium channel TREK-1 (Xi et al., 2011). Another recent report further suggested that direct modulation of the GR activity by anti-depressants underlies this beneficial effect of these compounds on hippocampal neurogenesis (Anacker et al., 2011). In fact, GR is known to be a direct target of anti-depressants in the treatment of depressive subjects (Pariante et al., 1997; Funato et al., 2006). Several previous studies demonstrated direct induction of GR nuclear translocation and suppression of its transcriptional activity by anti-depressants, although several opposing findings were reported (Bessa et al., 2009). In rats, lithium, a compound broadly used in the treatment of bipolar disorder, was also shown to stimulate hippocampal neurogenesis and neuronal differentiation (Chen et al., 2000). Interestingly, one *in vitro* study employing purified hippocampal progenitor cells demonstrated that lithium reverses glucocorticoid-induced suppression of progenitor cell proliferation, indicating the presence of crosstalk between lithium and glucocorticoids on hippocampal neurogenesis (Boku et al., 2009). Taken together, the above pieces of evidence point out that anti-depressants and lithium may improve mood of depressive subjects in part by altering hippocampal neurogenesis through attenuation of the negative effect of glucocorticoids on these cells, further suggesting contribution of stress- and glucocorticoid-mediated modulation of hippocampal progenitor cells in the development and/or pathophysiology of these mood disorders.

## Future perspectives

Discovery of adult neural stem cells and neural progenitor cells is a huge step forward in the field of neuroscience, as it has long been believed that the adult brain does not produce new neurons. Presence of adult neural progenitor cells has extended our understanding on the neural plasticity, as these cells play a central role in the adjustment of CNS functions to internal and external environmental changes. On the other hand, the HPA axis/stress system is the neuroendocrine system, which translates the various stressful signals or threats sensed by peripheral organs into a humoral signal “circulating glucocorticoid hormone” in order to adjust body's functions to cope with a new environment. Since neural progenitor cells residing in the hippocampal dentate gyrus participate in neuroplasticity (e.g., learning/memory and behavior/mood) of this brain area, it is quite reasonable that hippocampal progenitor cells and the HPA axis/glucocorticoids stress system cooperate with each other through their mutual and multi-level interactions. Indeed, hippocampal neurogenesis is critical for the hippocampus-mediated negative control on the HPA axis (Snyder et al., 2011), and glucocorticoids exert strong regulatory roles on hippocampal progenitor cells by changing their proliferation, differentiation, and/or cell fate (Schoenfeld and Gould, 2013). Although a broad overview of the interaction between these two systems has been revealed recently by using various animal and cellular systems, their detailed interaction is still missing, particularly those through the neurogenic niche of the hippocampus formed by cellular and humoral communications. In addition, the cellular mechanisms mediating glucocorticoid actions in hippocampal progenitor cells have not yet been fully elucidated. Finally, contribution of their interaction to the pathophysiology of cognitive and mood disorders should be verified clearly. Future intensive research will warrant these attractive research areas.

### Conflict of interest statement

The author declares that the research was conducted in the absence of any commercial or financial relationships that could be construed as a potential conflict of interest.
